# WHO FITS AND HOW? SUPPORTING TENANCY AND FOSTERING BELONGING IN HOUSING FOR OLDER PERSONS EXPERIENCING HOMELESSNESS

**DOI:** 10.1093/geroni/igae098.1106

**Published:** 2024-12-31

**Authors:** Lena Rebecca Richardson, Rachel Weldrick, Tam Perry

**Affiliations:** Chair; Co-Chair; Discussant

## Abstract

Homelessness among adults age 50+ years is increasing across North America. The Aging in the Right Place (AIRP) Partnership is a 5-year study of eleven promising practice organizations addressing older adult homelessness in three Canadian cities. This symposium will share findings from AIRP research on what supports a sense of belonging and social integration among older persons experiencing homelessness (OPEH) within temporary and permanent supportive housing programs. Presenters include interdisciplinary researchers with a diversity of perspectives stemming from gerontology, education, and social work. The symposium will begin with Erisman presenting a study of service providers’ perspectives on who is eligible for services and what actions providers can undertake to support clients’ ongoing tenancy in shelter/housing organizations. Weldrick will then discuss mechanisms for successfully supporting social integration from a study with OPEH in a temporary housing program. Following, Richardson will present findings on how multidirectional caring relations can foster belonging in a homeless shelter for older adults fleeing abuse. Finally, Walsh’s study findings will highlight ways to promote AIRP for male veterans in a temporary housing program. Tam Perry, an expert in housing transitions for older adults, will discuss the implications of these papers and reflect on processes of social integration and cultivating belonging for OPEH in temporary and permanent housing. Together, participants of the symposium will advance this emerging scholarship using a wide range of methods and perspectives.

